# Brain Structural and Functional Changes in Cognitive Impairment Due to Alzheimer’s Disease

**DOI:** 10.3389/fpsyg.2022.886619

**Published:** 2022-06-21

**Authors:** Marina Ávila-Villanueva, Alberto Marcos Dolado, Jaime Gómez-Ramírez, Miguel Fernández-Blázquez

**Affiliations:** ^1^Research in Alzheimer’s Disease, Departamento de Psicología Experimental, Procesos Cognitivos y Logopedia Universidad Complutense de Madrid (UCM), Madrid, Spain; ^2^Servicio de Neurología, Hospital Clínico San Carlos, Madrid, Spain; ^3^Instituto de Investigación Sanitaria del Hospital Clínico San Carlos, Madrid, Spain; ^4^Instituto de Investigación e Innovación Biomédica de Cádiz, Universidad de Cádiz, Cádiz, Spain; ^5^Departamento de Psicología Biológica y de la Salud, Facultad de Psicología, Universidad Autónoma de Madrid, Madrid, Spain

**Keywords:** structural changes, functional changes, early markers, reversion, cognitive impairment (CI)

## Abstract

Cognitive neuropsychology seeks a potential alignment between structural and functional brain features to explain physiological or pathological processes, such as Alzheimer’s disease (AD). Several structural and functional brain changes occurring during the disease, including cognitive impairment, are found at the end of the patient’s life, but we need to know more about what happens before its onset. In order to do that, we need earlier biomarkers at preclinical stages, defined by those biomarkers, to prevent the cognitive impairment. In this minireview, we have tried to describe the structural and functional changes found at different stages during AD, focusing on those features taking place before clinical diagnosis.

## Introduction

Alzheimer’s disease (AD) is the most prominent among neurodegenerative disorders, being responsible for structural and functional brain changes that may result in the functional changes that can be found during the development of the disease (Masters et al., 2015; Jack, et al., 2018).

We have an extensive knowledge of the features of the disease, but we are unable to cure or prevent it. Indeed, when a clear clinical diagnostic arrives, it is too late to prevent the disease, whose diagnosis is only definitely confirmed when the hallmarks of the disease are characterized at brain level in the autopsy after patients’ death, as firstly described by Alzheimer (1907) (Alzheimer et al., 1995).

Thus, we know several brain structural and functional changes occurring during the disease that are found only after the patient’s life, but we need to know more about what happens not only during the development and the end of the disease, but before its onset. In order to do that, we need earlier markers that could be obtained from liquid samples [blood or cerebrospinal fluid (CSF)] (Barthélemy et al., 2020) or image analysis like PET (Vlasenko et al., 2021), which in the case of tau pathology (Villemagne et al., 2015) can recapitulate Braak stages found in patients’ autopsies. These earlier markers, complemented with functional analysis, are needed to prevent the disorder.

In this minireview, we have tried to start describing the structural and functional brain changes found in the disease, and those features taking place before clinic diagnosis.

We also comment on the possible alignment between structural and functional brain changes that may occur at different times in the life of a human being, that could end in dementia. Many structures can be involved in AD development. Indeed, brain regions involved in the Default Mode Network (DMN) may play a role (Alves et al., 2019) at different time and levels.

We propose, as it has been indicated by others, that long longitudinal studies to follow those possible changes several years before dementia onset will facilitate the step-by-step analysis of AD development. Since some of those analyses have already been carried out, we will comment on some of the obtained results looking at structural and functional time-dependent brain changes.

## Structural Changes Related to AD Dementia

As indicated by Alzheimer et al. (1995), the presence of two main aberrant structures, senile plaques and neurofibrillary tangles, precede the neuronal death and brain degeneration found later on the disease. Plaques are aggregates of beta amyloid peptide that progressively appear during the development of the disease following a specific pattern (Thal et al., 2002). This pattern starts in the neocortex and continues through the allocortex, hippocampus, basal ganglia, midbrain, and cerebellum. This pathway starts many years before clinical diagnostic (Bateman et al., 2012). Later on, it can be found by the presence of neurofibrillary tangles (tau protein aggregates), that also propagate, but follow a different pathway (Braak and Braak, 1991), starting at the transentorhinal region and expanding through the entorhinal cortex, hippocampus and neocortical areas. In this way, there are two different pathologies (amyloid and tau pathologies, with different structural changes) that appear at different times during the development of the disease. Indeed, it has been suggested that the disorder can become a unique disease when both amyloid and tau pathologies overlap (Hojjati et al., 2021). Before that time, different features may be related to the presence of only plaques in some brain regions.

## Functional Changes Related to AD Dementia

Memory loss, cognitive impairment, loss of executive functions, and loss of consciousness, among others (Masters et al., 2015; Jack, et al., 2018), occur in AD dementia. They can appear at different times during disease development, step by step through a continuum that ends in dementia and an extensive brain degeneration. Being aware of one’s surroundings and considering social behavior as part of the interaction with that environment, it is important to note that it changes with aging (Rosati et al., 2020), which is relevant taking into account that aging itself constitutes the main risk for dementia. Indeed, there is a loss of consciousness, related to loss of individual awareness or awareness related to the world around those future patients.

## Mild Cognitive Impairment

We now know that before dementia there is a mild cognitive impairment (MCI), that could be more related to tau pathology. At the end of the past century (Petersen et al., 1999), it was suggested that before dementia and probably related to the onset of the first Braak stages, MCI could result in changes in cognition, while maintaining the capacity for executive functions and the independence to carry out daily activities. MCI definition can be classified into two different types, amnesic and non-amnesic. The first one is more related to memory changes, the second one may maintain an intact executive functionality (Carmasin et al., 2021). Moreover, MCI could be related to Braak stages 1 and 2 and CA1 region could be involved in the appearance of MCI.

The existence of a so-called AD continuum could indicate the presence of MCI before AD (Petersen et al., 1999). Although there are several types of MCI, including amnestic, non-amnestic, and mixed, that could behave differently to progress into dementia, we will mainly comment on MCI as a whole.

Around 10–15% of subjects with MCI could progress to dementia per year (Petersen, 2000) and it has been estimated that overall more than 40% of subjects with MCI could develop dementia (Panpalli Ates and Yilmaz Can, 2020). Thus, it is paramount to know why the other 60% do not progress similarly.

The percentage of transition from MCI to dementia depends on factors like age, education, family history of dementia, vascular risk factors or ApoE4 status (Kryscio et al., 2006). Also, lifestyle-related factors like alcohol consumption have a role on this proportion (Xu et al., 2009). Some of these factors could be modified to prevent the development of the disease (Sanz-Blasco et al., 2021).

## Subjective Cognitive Decline

It has been suggested that subjective cognitive decline (SCD), expressed by a frequent confusion and transitory memory loss could be a cognitive decline without being an objective (testable) mild cognitive impairment. Thus, it has been suggested that SCD could be a previous step to MCI (Jessen et al., 2014).

## Transition From Subjective Cognitive Decline to Mild Cognitive Impairment

In a 7-year longitudinal study, it was described that around 20% of SCD subjects can progress to MCI (Bessi et al., 2018). Similar results were found in other studies (Avila-Villanueva and Fernandez-Blazquez, 2017).

Thus, SCD is a clear risk factor for MCI, like MCI is a risk factor for dementia.

Again, it will be of interest to identify the causes for the transition of that 20% SCD subjects to MCI, looking for a possible prevention.

## Executive Functions

In addition to episodic memory loss, related to changes in the CA1 hippocampal region, a main feature on the development of AD is the loss of executive functions, like planning, working memory, self-control, flexible thinking, or organization. Executive functions have been mainly located in prefrontal regions (Stuss, 2011), although other regions like nucleus accumbens (NAcc) could also play a role in such functions (Floresco, 2015; Prasad, 2018). More recently, this role has been proposed again (Jenkins et al., 2021). Since hippocampal CA1 can connect to NAcc (Zhou et al., 2020), a damage in CA1 could later-on have an effect on NAcc and on executive functions or specific types of memories (Prasad, 2018).

In this way, it will be of interest to know if some features of cognitive decline related to CA1 damage could take place, or not, earlier than those specific executive functions. Further analysis should be done to test if it is the case.

## Is There a Change Before Subjective Cognitive Decline? Could Be That Change Chronic Stress

We have previously discussed the role of chronic stress as a trigger for the AD continuum, being a possible step before SCD (Avila-Villanueva et al., 2020).

Structurally, chronic stress may affect structures like amygdala (Liu et al., 2020). Amygdala could activate other brain areas, such as hypothalamus and brainstem, altering prefrontal cortex (PFC) function (Arnsten, 2009). Also, chronic stress may induce changes in the sympathetic nervous system altering the hypothalamic-pituitary-adrenal axis and producing an increase of cortisol, a compound that can cross the brain-blood barrier and is able to bind to hippocampus, amygdala, or prefrontal cortex receptors (de Kloet et al., 1999; Li et al., 2019). Thus, damage in those structures may be a previous step to SCD. Indeed, people with chronic stress in midlife could have a higher risk for SCD and MCI (Avila-Villanueva et al., 2020). This also agrees well with the fact that subjects with SCD tend to have a higher level of cortisol, a marker for chronic stress (Fiocco et al., 2006).

Additionally, depression or anxiety could be functional factors, taking place before MCI or dementia and they may correlate with changes in structural areas like amygdala (Liu et al., 2020). Chronic stress in turn can be consequence of the lifestyle, being poverty, the main cause of chronic stress (Fernandez-Blazquez et al., 2021). On the other hand, cortisol secretion is linked to circadian rhythm and a relation between sleeping time, cortisol secretion, and dementia has been recently indicated (Antypa et al., 2021).

## Consciousness and “Hidden” Structures That Could Be Involved Before the Appearance of Cognitive Impairment

**FIGURE 1:**
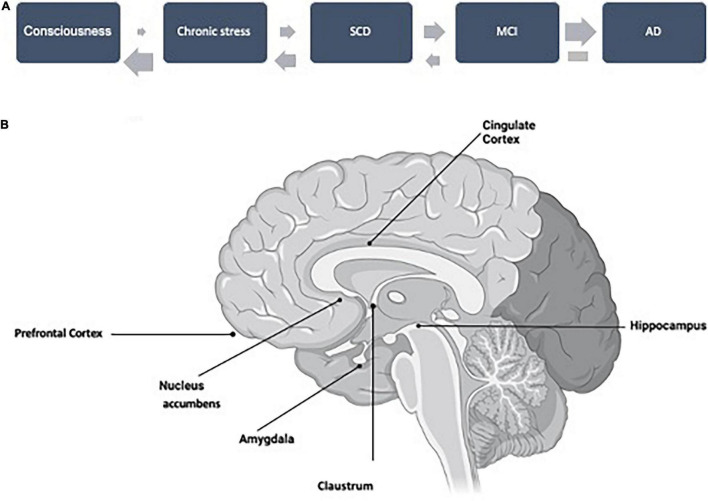
**(A)** A working hypothesis for the AD continuum, starting with a putative change in consciousness, related to amyloid pathology, followed by features that could facilitate the development of the continuum like chronic stress, SCD, and MCI. The probability of transition (upper arrows) is increasing step by step. These steps could be reverted but not that from AD (dementia), with an inversion probability (lower arrows). **(B)** Profile of an open brain sagittal section indicating the location of the brain structures mentioned throughout this minireview.

In Familial Alzheimer Disease (FAD), consciousness changes have been considered as an early marker of the disease (Aschenbrenner et al., 2020), and claustrum has been proposed to be a brain area controlling consciousness (Crick and Koch, 2003). Claustrum is a “hidden” structure, located below the insula cortex, that can only be visualized when other parts of the cortex are pulled aside. Claustrum dysfunction may precede amyloid accumulation and aggregation in FAD (Goutagny et al., 2013). Additionally, claustrum can establish connections with entorhinal cortex (Kurada et al., 2019) and hippocampal areas (Amaral and Cowan, 1980), which have been related to tau pathology, and cognitive impairment. Thus, we suggest that further studies analyzing the possible role of claustrum in very early stages of AD should be performed, not only on FAD, but also in sporadic Alzheimer’s disease (SAD). If there is a role of claustrum in SAD, a very early functional change in the AD continuum could be related to controlling consciousness (Figure 1A).

## Reversion in the AD Continuum

In this commentary, we have suggested the possible AD continuum shown in Figure 1, but we would like to discuss whether it is unidirectional in all steps or it could be bidirectional in some of them (Figure 1A). We know, that it is not possible to revert dementia, but subjects from the previous step, MCI, could revert to normal condition in a significant proportion (Sanz-Blasco et al., 2021).

A high proportion of MCI subjects can progress to dementia. Curiously, a similar proportion could revert to a normal cognitive situation (Sanz-Blasco et al., 2021). Some features that could be involved in one or the opposite direction have already been suggested (Sanz-Blasco et al., 2021), but further studies should be carried out. One example could be that circadian-rest activity could predict cognitive decline in MCI subjects (Targa et al., 2021). Additionally, a good characterization of previous stages of MCI (like SCD), of MCI itself, and dementia should be carried out [see for example Yang et al. (2021)], to accurately determine the transition among stages. On the other hand, it should be discussed that psychological tests are more focused on changes in cognitive or executive dysfunctions than changes in emotional or social behavior. In this way, it can be proposed that for AD development, at very early steps, Aß and tau pathologies could follow different structural and functional pathways, being those of Aß pathology, probably, more related to emotions (less present in clinical tests) whereas tau pathology is more related to cognitive impairment or memory decrease. Accordingly, Figure 1B shows a possible relation between specific structural changes with specific functional (cognitive, behavioral, or emotional) changes.

## Reversion From Mild Cognitive Impairment to Normal Cognition

Recently, it has been shown that the likelihood of progression from MCI to dementia is very similar to the reversion from MCI to normal cognition (Sanz-Blasco et al., 2021). Some factors involved in that reversion have been described (Sanz-Blasco et al., 2021), but there are other features that should be analyzed, based on the previous history of the patient. For instance, we have previously commented on this minireview the possible role of chronic stress as a very early risk factor for dementia. Suitably, reversion of chronic stress correlates with reversion to a normal healthy cognition. However, in some cases that stress results in the irreversible atrophy of dentate gyrus neurons (Bai et al., 2012), which remains as a risk signature that could facilitate the future progression to dementia. In addition, morphological (unreversible?) reorganization, in hippocampus, nucleus accumbens, and amygdala has been reported after corticosterone administration (Morales-Medina et al., 2009).

In this way, we would like to comment that new psychological tests to determine changes in social and emotional behavior may be needed to account for possible changes related to the presence of amyloid plaques at specific brain locations at very early times of the continuum. Recently, changes in emotion and generosity in older adults have been reported (Carstensen and Chi, 2021), but little was done in MCI/AD patients, especially at early stages of the disorder.

In humans, social and emotional processing is mainly localized at cerebral neocortex in areas like the orbital frontal cortex. In the first Thal stage, this area already shows an evident amount of amyloid aggregation. Although it has been reported that orbital frontal cortex is involved in emotional enhancement of memory (Kumfor et al., 2013), there are not many studies looking for possible behavioral changes at those early Thal stages. Curiously, orbital frontal cortex pathology related to AD has been probably more analyzed by examining at tau pathology than amyloid pathology (Tekin et al., 2001). It has also been indicated that damage on the orbitofrontal cortex and the anterior cingulate cortex correlate with behavioral changes, for example dealing with empathy (Avila-Villanueva et al., 2021). Thus, we suggest the possibility of preparing behavioral psychological tests to explore changes at very early timepoints of the continuum. These tests may analyze behavioral changes, expressed by symptoms like agitation, disinhibition, elation, anxiety, or depression (Cajanus et al., 2019), and other features related to subjective wellbeing (van Zonneveld, 1961), similar to EuroQoL-5D (Dolan, 1997). These tests could be a good complement for the previous ones measuring memory changes, cognition, or executive functions, more related to the development of tau pathology, even though during the development of the disease there is an overlapping of both pathologies. Indeed, there such overlapping during the spreading of tau pathology is linked to the fact that tau spreading is favored by the presence of amyloid aggregates (Busche and Hyman, 2020). This tau spreading from the hippocampal area to cerebral cortex is related to an initial memory (and cognitive) impairment, which endpoint could be dementia. Thus, in Figure 1B, we have shown a relation between structural changes (with the presence of amyloid, tau, or both pathologies) with specific functional (behavioral, emotional, memory, or cognitive) changes, occurring at different times of the AD continuum.

## Conclusion

We propose that further analysis should focus on indicating the features that favor the progression into dementia or those that could be involved in the reversion to a normal cognitive situation (Sanz-Blasco et al., 2021). The knowledge of the later features may facilitate the use of therapeutical tools at very early stages of the AD continuum. The possible reversion will probably need some extent of in-depth knowledge of the patient through the recently proposed precision (personalized) medicine (Hampel et al., 2018, 2020) and also by taking into account the possible correlation between image studies to analyze structural changes with functional failures analyzed by neurological and neuropsychological studies.

## Author Contributions

All authors were responsible for the conceptualization, reviewing the literature, and critically editing the manuscript, approved the submitted version of the manuscript, and were accountable for the accuracy and integrity of the work.

## Conflict of Interest

The authors declare that the research was conducted in the absence of any commercial or financial relationships that could be construed as a potential conflict of interest.

## Publisher’s Note

All claims expressed in this article are solely those of the authors and do not necessarily represent those of their affiliated organizations, or those of the publisher, the editors and the reviewers. Any product that may be evaluated in this article, or claim that may be made by its manufacturer, is not guaranteed or endorsed by the publisher.

## References

[B1] Alves P. N. Foulon C. Karolis V. Bzdok D. Margulies D. S. Volle E. (2019). An improved neuroanatomical model of the default-mode network reconciles previous neuroimaging and neuropathological findings. *Commun. Biol.* 2:370. 10.1038/s42003-019-0611-3 31633061 PMC6787009

[B2] Alzheimer A. Stelzmann R. A. Schnitzlein H. N. Murtagh F. R. (1995). An english translation of alzheimer’s 1907 paper, “uber eine eigenartige erkankung der hirnrinde”. *Clin. Anat.* 8 429–431. 10.1002/ca.980080612 8713166

[B3] Amaral D. G. Cowan W. M. (1980). Subcortical afferents to the hippocampal formation in the monkey. *J. Comp. Neurol.* 189 573–591. 10.1002/cne.901890402 6769979

[B4] Antypa D. Perrault A. A. Vuilleumier P. Schwartz S. Rimmele U. (2021). Suppressing the morning cortisol rise after memory reactivation at 4 a.m. enhances episodic memory reconsolidation in humans. *J. Neurosci.* 41 7259–7266. 10.1523/JNEUROSCI.0096-21.2021 34266897 PMC8387108

[B5] Arnsten A. F. (2009). Stress signalling pathways that impair prefrontal cortex structure and function. *Nat. Rev. Neurosci.* 10 410–422. 10.1038/nrn2648 19455173 PMC2907136

[B6] Aschenbrenner A. J. James B. D. McDade E. Wang G. Lim Y. Y. Benzinger T. L. S. (2020). Awareness of genetic risk in the dominantly inherited alzheimer network (DIAN). *Alzheimers Dement.* 16 219–228. 10.1002/alz.12010 31914221 PMC7206736

[B7] Avila-Villanueva M. Fernandez-Blazquez M. A. (2017). Subjective cognitive decline as a preclinical marker for alzheimer’s disease: the challenge of stability over time. *Front. Aging Neurosci.* 9:377. 10.3389/fnagi.2017.00377 29201004 PMC5696596

[B8] Avila-Villanueva M. Gomez-Ramirez J. Avila J. Fernandez-Blazquez M. A. (2021). Alzheimer’s disease and empathic abilities: the proposed role of the cingulate cortex. *J. Alzheimers Dis. Rep.* 5 345–352. 10.3233/ADR-200282 34189406 PMC8203285

[B9] Avila-Villanueva M. Gomez-Ramirez J. Maestu F. Venero C. Avila J. Fernandez-Blazquez M. A. (2020). The role of chronic stress as a trigger for the alzheimer disease continuum. *Front. Aging Neurosci.* 12:561504. 10.3389/fnagi.2020.561504 33192456 PMC7642953

[B10] Bai M. Zhu X. Zhang Y. Zhang S. Zhang L. Xue L. (2012). Abnormal hippocampal BDNF and miR-16 expression is associated with depression-like behaviors induced by stress during early life. *PLoS One* 7:e46921. 10.1371/journal.pone.0046921 23056528 PMC3466179

[B11] Barthélemy N. R. Horie K. Sato C. Bateman R. J. (2020). Blood plasma phosphorylated-tau isoforms track CNS change in Alzheimer’s disease. *J. Exp. Med.* 217:e20200861. 10.1084/jem.20200861 32725127 PMC7596823

[B12] Bateman R. J. Xiong C. Benzinger T. L. Fagan A. M. Goate A. Fox N. C. (2012). Clinical and biomarker changes in dominantly inherited Alzheimer’s disease. *N. Engl. J. Med.* 367 795–804. 10.1056/NEJMoa1202753 22784036 PMC3474597

[B13] Bessi V. Mazzeo S. Padiglioni S. Piccini C. Nacmias B. Sorbi S. (2018). From subjective cognitive decline to alzheimer’s disease: the predictive role of neuropsychological assessment, personality traits, and cognitive reserve. a 7-year follow-up study. *J. Alzheimers Dis.* 63 1523–1535. 10.3233/JAD-171180 29782316

[B14] Braak H. Braak E. (1991). Neuropathological stageing of Alzheimer-related changes. *Acta Neuropathol.* 82 239–259. 10.1007/BF00308809 1759558

[B15] Busche M. A. Hyman B. T. (2020). Synergy between amyloid-beta and tau in Alzheimer’s disease. *Nat. Neurosci.* 23 1183–1193. 32778792 10.1038/s41593-020-0687-6 PMC11831977

[B16] Cajanus A. Solje E. Koikkalainen J. Lotjonen J. Suhonen N. M. Hallikainen I. (2019). The association between distinct frontal brain volumes and behavioral symptoms in mild cognitive impairment, alzheimer’s disease, and frontotemporal dementia. *Front. Neurol.* 10:1059. 10.3389/fneur.2019.01059 31632342 PMC6786130

[B17] Carmasin J. S. Roth R. M. Rabin L. A. Englert J. J. Flashman L. A. Saykin A. J. (2021). Stability of subjective executive functioning in older adults with aMCI and subjective cognitive decline. *Arch. Clin. Neuropsychol.* 36, 1012–1018. 33454755 10.1093/arclin/acaa129 PMC8406646

[B18] Carstensen L. L. Chi K. (2021). Emotion and prosocial giving in older adults. *Nat. Aging* 1 866–867. 10.1038/s43587-021-00126-3 35128464 PMC8813056

[B19] Crick F. Koch C. (2003). A framework for consciousness. *Nat. Neurosci.* 6 119–126. 12555104 10.1038/nn0203-119

[B20] de Kloet E. R. Oitzl M. S. Joels M. (1999). Stress and cognition: are corticosteroids good or bad guys? *Trends Neurosci.* 22 422–426. 10.1016/s0166-2236(99)01438-1 10481183

[B21] Dolan P. (1997). Modeling valuations for EuroQol health states. *Med. Care* 35 1095–1108. 9366889 10.1097/00005650-199711000-00002

[B22] Fernandez-Blazquez M. A. Noriega-Ruiz B. Avila-Villanueva M. Valenti-Soler M. Frades-Payo B. Del Ser T. (2021). Impact of individual and neighborhood dimensions of socioeconomic status on the prevalence of mild cognitive impairment over seven-year follow-up. *Aging Ment. Health* 25 814–823. 10.1080/13607863.2020.1725803 32067489

[B23] Fiocco A. J. Wan N. Weekes N. Pim H. Lupien S. J. (2006). Diurnal cycle of salivary cortisol in older adult men and women with subjective complaints of memory deficits and/or depressive symptoms: relation to cognitive functioning. *Stress* 9 143–152. 10.1080/10253890600965674 17060048

[B24] Floresco S. B. (2015). The nucleus accumbens: an interface between cognition, emotion, and action. *Annu. Rev. Psychol.* 66 25–52. 10.1146/annurev-psych-010213-115159 25251489

[B25] Goutagny R. Gu N. Cavanagh C. Jackson J. Chabot J. G. Quirion R. (2013). Alterations in hippocampal network oscillations and theta-gamma coupling arise before Abeta overproduction in a mouse model of Alzheimer’s disease. *Eur. J. Neurosci.* 37 1896–1902. 10.1111/ejn.12233 23773058

[B26] Hampel H. Caraci F. Cuello A. C. Caruso G. Nistico R. Corbo M. (2020). A path toward precision medicine for neuroinflammatory mechanisms in alzheimer’s disease. *Front. Immunol.* 11:456. 10.3389/fimmu.2020.00456 32296418 PMC7137904

[B27] Hampel H. Toschi N. Babiloni C. Baldacci F. Black K. L. Bokde A. L. W. (2018). Revolution of alzheimer precision neurology. passageway of systems biology and neurophysiology. *J. Alzheimers Dis.* 64 S47–S105. 10.3233/JAD-179932 29562524 PMC6008221

[B28] Hojjati S. H. Feiz F. Ozoria S. Razlighi Q. R., and Alzheimer’s Disease Neuroimaging Initiative (2021). Topographical overlapping of the amyloid-beta and tau pathologies in the default mode network predicts alzheimer’s disease with higher specificity. *J. Alzheimers Dis.* 83 407–421. 10.3233/JAD-210419 34219729

[B29] Jack C. R. Bennett D. A. Blennow K. Carrillo M. C. Dunn B. Haeberlein S. B. (2018). NIA-AA research framework: toward a biological definition of alzheimer’s disease. *Alzheimers Dement.* 14 535–562. 10.1016/j.jalz.2018.02.018 29653606 PMC5958625

[B30] Jenkins L. M. Kogan A. Malinab M. Ingo C. Sedaghat S. Bryan N. R. (2021). Blood pressure, executive function, and network connectivity in middle-aged adults at risk of dementia in late life. *Proc. Natl. Acad. Sci. U.S.A.* 118:e2024265118. 10.1073/pnas.2024265118 34493658 PMC8449402

[B31] Jessen F. Amariglio R. E. van Boxtel M. Breteler M. Ceccaldi M. Chetelat G. (2014). A conceptual framework for research on subjective cognitive decline in preclinical Alzheimer’s disease. *Alzheimers Dement.* 10 844–852. 10.1016/j.jalz.2014.01.001 24798886 PMC4317324

[B32] Kryscio R. J. Schmitt F. A. Salazar J. C. Mendiondo M. S. Markesbery W. R. (2006). Risk factors for transitions from normal to mild cognitive impairment and dementia. *Neurology* 66 828–832. 10.1212/01.wnl.0000203264.71880.45 16567698

[B33] Kumfor F. Irish M. Hodges J. R. Piguet O. (2013). The orbitofrontal cortex is involved in emotional enhancement of memory: evidence from the dementias. *Brain* 136 2992–3003. 10.1093/brain/awt185 23838694

[B34] Kurada L. Bayat A. Joshi S. Koubeissi M. Z. (2019). The claustrum in relation to seizures and electrical stimulation. *Front. Neuroanat.* 13:8. 10.3389/fnana.2019.00008 30809132 PMC6379271

[B35] Li Y. Qin J. Yan J. Zhang N. Xu Y. Zhu Y. (2019). Differences of physical vs. psychological stress: evidences from glucocorticoid receptor expression, hippocampal subfields injury, and behavioral abnormalities. *Brain Imaging Behav.* 13 1780–1788. 10.1007/s11682-018-9956-3 30229371

[B36] Liu W. Z. Zhang W. H. Zheng Z. H. Zou J. X. Liu X. X. Huang S. H. (2020). Identification of a prefrontal cortex-to-amygdala pathway for chronic stress-induced anxiety. *Nat. Commun.* 11:2221. 10.1038/s41467-020-15920-7 32376858 PMC7203160

[B37] Masters C. L. Bateman R. Blennow K. Rowe C. C. Sperling R. A. Cummings J. L. (2015). Alzheimer’s disease. *Nat. Rev. Dis. Primers* 1:15056. 10.1038/nrdp.2015.56 27188934

[B38] Morales-Medina J. C. Sanchez F. Flores G. Dumont Y. Quirion R. (2009). Morphological reorganization after repeated corticosterone administration in the hippocampus, nucleus accumbens and amygdala in the rat. *J. Chem. Neuroanat.* 38 266–272. 10.1016/j.jchemneu.2009.05.009 19505571

[B39] Panpalli Ates M. Yilmaz Can F. (2020). Which factors can we control the transition from mild cognitive impairment to dementia? *J. Clin. Neurosci.* 73 108–110. 10.1016/j.jocn.2020.01.015 31992514

[B40] Petersen R. C. (2000). Mild cognitive impairment: transition between aging and Alzheimer’s disease. *Neurologia* 15 93–101. 10846869

[B41] Petersen R. C. Smith G. E. Waring S. C. Ivnik R. J. Tangalos E. G. Kokmen E. (1999). Mild cognitive impairment: clinical characterization and outcome. *Arch. Neurol.* 56 303–308. 10.1001/archneur.56.3.303 10190820

[B42] Prasad J. A. (2018). Exploring executive functions using a distributed circuit model. *J. Neurosci.* 38 5039–5041. 10.1523/JNEUROSCI.0549-18.2018 29848624 PMC6705944

[B43] Rosati A. G. Hagberg L. Enigk D. K. Otali E. Emery Thompson M. Muller M. N. (2020). Social selectivity in aging wild chimpanzees. *Science* 370, 473–476. 10.1126/science.aaz9129 33093111 PMC7668794

[B44] Sanz-Blasco R. Ruiz-Sanchez de Leon J. M. Avila-Villanueva M. Valenti-Soler M. Gomez-Ramirez J. Fernandez-Blazquez M. A. (2021). Transition from mild cognitive impairment to normal cognition: determining the predictors of reversion with multi-state Markov models. *Alzheimers Dement.* 10.1002/alz.12448 34482637

[B45] Stuss D. T. (2011). Functions of the frontal lobes: relation to executive functions. *J. Int. Neuropsychol. Soc.* 17 759–765. 10.1017/S1355617711000695 21729406

[B46] Targa A. D. S. Benitez I. D. Dakterzada F. Fontenele-Araujo J. Minguez O. Zetterberg H. (2021). The circadian rest-activity pattern predicts cognitive decline among mild-moderate Alzheimer’s disease patients. *Alzheimers Res. Ther.* 13:161. 10.1186/s13195-021-00903-7 34563258 PMC8466995

[B47] Tekin S. Mega M. S. Masterman D. M. Chow T. Garakian J. Vinters H. V. (2001). Orbitofrontal and anterior cingulate cortex neurofibrillary tangle burden is associated with agitation in Alzheimer disease. *Ann. Neurol.* 49 355–361. 11261510

[B48] Thal D. R. Rub U. Orantes M. Braak H. (2002). Phases of A beta-deposition in the human brain and its relevance for the development of AD. *Neurology* 58 1791–1800. 10.1212/wnl.58.12.1791 12084879

[B49] van Zonneveld R. J. (1961). *The Health Of The Aged.* Assen: Van Gorcum.

[B54] Villemagne V. L. Fodero-Tavoletti M. T. Masters C. L. Rowe C. C. (2015). Tau imaging: early progress and future directions. *Lancet Neurol.* 14, 114–124. 10.1016/S1474-4422(14)70252-2 25496902

[B50] Vlasenko V. V. Rogers E. G. Waugh C. E. (2021). Affect labelling increases the intensity of positive emotions. *Cogn. Emot.* 35, 1350–1364. 10.1080/02699931.2021.195930 34323172

[B51] Xu G. Liu X. Yin Q. Zhu W. Zhang R. Fan X. (2009). Alcohol consumption and transition of mild cognitive impairment to dementia. *Psychiatry Clin. Neurosci.* 63 43–49. 10.1111/j.1440-1819.2008.01904.x 19154211

[B52] Yang Y. W. Hsu K. C. Wei C. Y. Tzeng R. C. Chiu P. Y. (2021). Operational determination of subjective cognitive decline, mild cognitive impairment, and dementia using sum of boxes of the clinical dementia rating scale. *Front. Aging Neurosci.* 13:705782. 10.3389/fnagi.2021.705782 34557083 PMC8455062

[B53] Zhou Y. Yan E. Cheng D. Zhu H. Liu Z. Chen X. (2020). The projection from ventral ca1, not prefrontal cortex, to nucleus accumbens core mediates recent memory retrieval of cocaine-conditioned place preference. *Front. Behav. Neurosci.* 14:558074. 10.3389/fnbeh.2020.558074 33304246 PMC7701212

